# Profound Near Fatal Respiratory Dysfunction in a Neonate With Meningomyelocele: A Narrative With Neurosurgical Lessons

**DOI:** 10.1155/crpe/5569749

**Published:** 2025-07-26

**Authors:** P. Singh, P. Kadam, K. Aniruddhan, C. Kaliaperumal

**Affiliations:** ^1^Department of Clinical Neurosciences, Royal Infirmary of Edinburgh, Edinburgh, UK; ^2^Department of Paediatrics, Victoria Hospital, Kirkcaldy, UK; ^3^Department of Neurosurgery, Royal Hospital for Children and Young People, Edinburgh, UK

**Keywords:** Arnold–Chiari malformation, autonomic nervous system, Chiari II malformation, meningomyelocele, paediatric neurosurgery

## Abstract

The association between spina bifida, specifically myelomeningocele, and autonomic dysfunction is known although rare. This case highlights the severe respiratory compromise that can occur in paediatric patients secondary to myelomeningocele. We describe a case of a neonate who experienced profound respiratory dysfunction following a successful lumbosacral myelomeningocele repair on Day 1 of life, on the background of a prenatal diagnosis of Arnold–Chiari Type II malformation and congenital hydrocephalus. In addition, the patient was found to have an incidental choroid plexus papilloma which was resected along with foramen magnum decompression. Even after multiple successful neurosurgical interventions, the patient experienced a number of apnoeic episodes requiring intubation and ventilation and a prolonged intensive care unit stay. Despite the exceptionally high frequency and severity of the apnoeic–hypoxic episodes, the patient is demonstrating age-appropriate cognitive development and is now ventilated overnight via tracheostomy. There are lessons to be learnt surrounding the multidisciplinary care of these patients, management of respiratory distress, and the different aetiologies in this case. There was also a correlation found between episodes of urinary tract infection and increased frequency of apnoeic episodes, raising the question of whether her bladder irritation may be partially triggering her autonomic dysreflexia, on a background of a significant surgical history.

**Summary**

• Established facts.
◦ There is a rare association between myelomeningocele and autonomic dysfunction, but little is documented about the management of such cases.
• Novel insights.
◦ Severe, near fatal respiratory dysfunction can occur secondary to myelomeningocele, and various causes of this are explored.
◦ Infection was noted to act as a trigger for episodes of respiratory apnoea in this patient.

## 1. Introduction

Whilst spina bifida is a relatively well-studied area of paediatric pathology, its association with autonomic dysfunction is much less well understood. There is a paucity of evidence in the literature to inform clinicians on outcomes for spina bifida patients with autonomic dysfunction of any nature due to the rarity of the occurrence, and as with this case, this includes respiratory dysreflexia.

The autonomic centres of the brain, namely, the respiratory and cardiac centres, lie in the medulla oblongata, a part of the brainstem. Autonomic centres are particularly vulnerable to trauma and changes in blood flow via vertebral arteries [1] and their tributaries and compression of the surrounding area of the fourth ventricle and the craniocervical junction. Interestingly, compression of the fourth ventricle is currently a practiced osteopathic technique, and although few RCTs have tested the technique, it is reported that this cranial manipulation may modify blood pressure, heart rate and autonomic function [2]. In particular, a mix of diffuse and focal injuries for individuals surviving traumatic brain injuries can affect autonomic function. Medical causes of autonomic dysfunction include metabolic issues such as diabetes and viruses such as HIV or Lyme disease [3, 4].

Management of mild anxiety and panic attacks, treated as another form of autonomic dysfunctional symptoms, has led practitioners to prescribe breathing techniques in order to reduce the adverse effects of chronic stress [5]. The techniques have lent themselves to the practice of mindfulness and have their roots in meditation and ancient Ayurvedic medicine from India [6].

Due to our case having multiple pathologies, not only the one, there may be more than one reason for the autonomic dysfunction. The hydrocephalus may well have been the result of the Chiari II malformation and an incidental choroid plexus papilloma, both causing obstruction to the CSF flow either internally or externally to the fourth ventricle. The Chiari II malformation may have resulted in cranial nerve dysfunction and disordered breathing due to brainstem compression. The compression of the superior aspect of the brainstem from the multiple pathologies including the choroid plexus papilloma may have accumulated to create the multiple apnoeic episodes.

The multiple small surgical interventions around the site of the craniocervical junction at the early stages of life may well have been associated with the consequences of autonomic dysfunction. The timing of the surgeries on one hand was essential and therefore it is difficult to speculate whether the respiratory sequalae may have been any different had the surgeries taken place later.

## 2. Case Presentation

**Figure 1:**
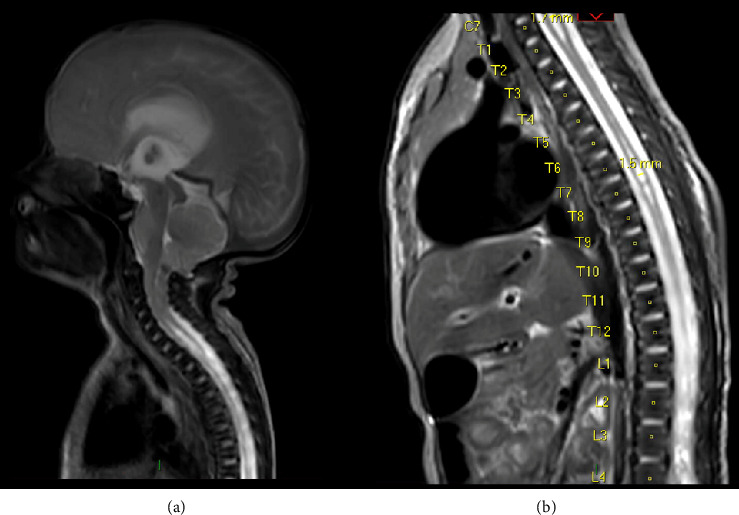
Preoperative T2 weighted sagittal MRI scan demonstrating (a) Chiari II malformation with cervical syringomyelia; (b) cervical and thoracic syringomyelia (from patient notes).

The case is of an infant born at 33 + 4 weeks known to have a prenatal diagnosis of spina bifida and Arnold–Chiari Type II malformation (Figure 1). In addition to the myelomeningocele repair, the patient underwent a ventriculoperitoneal shunt intervention to treat hydrocephalus and apnoeic episodes which were complications of the myelomeningocele repair.

**Figure 2:**
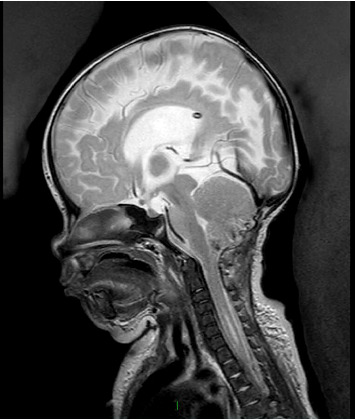
Three-month postoperative T2 weighted sagittal MRI demonstrating resection of the tumour and improvement in cervical syringomyelia (from patient notes).

**Figure 3:**
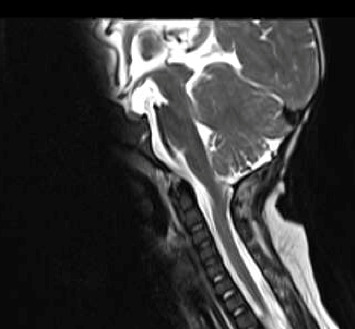
One-year postoperative T2 weighted sagittal MRI demonstrating complete resolution of cervical syringomyelia (from patient notes).

The patient struggled with maintaining her oxygen saturations and required nocturnal BiPAP. An MRI head performed due to persistent apnoeic episodes revealed tonsillar descent to T4, merging with a thoracic syrinx ending at T7 from C5. Later, the patient underwent foramen magnum decompression to treat these pathologies, which was thought to be contributing or the cause of the apnoeic episodes. During the surgery, a pale lesion in the fourth ventricle was found incidentally near the craniocervical junction and resected (Figures 2 and 3). This was histologically diagnosed as a Grade 1 choroid plexus papilloma. No chromosomal abnormalities were found with clinical genetics testing.

**Figure 4:**
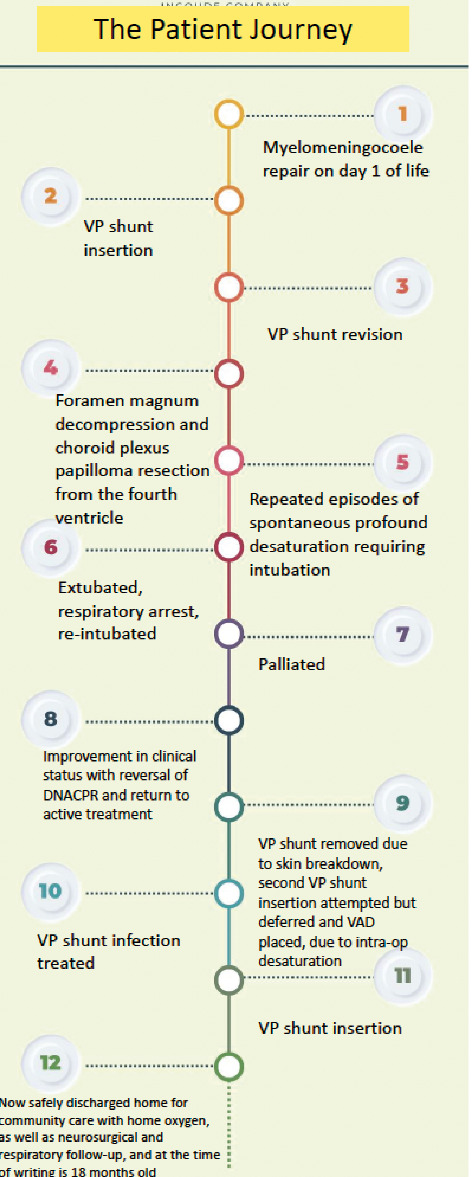
A timeline of surgical interventions from Day 1 of life to discharge home (own work).

Despite both the respiratory management and neurosurgical interventions, she continued to suffer cyanotic episodes and respiratory arrests. She became dependent on a ventilator in the intensive care unit for a short time and was given a palliative diagnosis. Although the situation was distressing and had moments of bleakness, a strong multidisciplinary teams pulled together in their collaborative efforts to treat the patient, particularly around the decision to wean from the ventilator and to give the patient a chance with the home oxygen (Figure 4).

The swift decision-making from neurosurgery, performing procedures in a short space of time also reflects the swift discussion and planning within the team. At the time of this writing, the patient, 18 months old and ventilated via tracheostomy at night, is preparing to go home under the care of her parents.

Interestingly, paediatric sleep studies found that the apnoeic episodes which the patient experiences were consistent with severe rapid eye movement (REM) sleep disordered breathing [7].

### 2.1. Reflections on Decision-Making

When the apnoeic episodes increased in frequency postneurosurgery, which was done for foramen magnum decompression and choroid plexus papilloma resection, the management of the patient changed and paediatric respiratory specialists began to discuss with neurosurgery about her prognosis. She became cyanosed and less responsive with a profound near fatal respiratory arrest. The lowest SpO_2_ recorded was 9% along with severe hypotension. Due to these events, neonatal and respiratory specialists made a decision to intubate under guidance by intensive care unit colleagues. The parents of the patient were counselled as to the need for ventilatory support and the sequalae which had led to this decision. After gaining consent from her parents, she was intubated and ventilated to support her respiratory needs whilst giving her time to recover.

Her trajectory changed when the patient was found to not be improving and becoming dependent on the ventilatory support. Her high baseline PaCO_2_ of 7.02 coupled with poorly synchronised desaturations to ventilator-assisted support was consistent with underlying brainstem dysfunction. Due to these events, she was given a palliative diagnosis with a DNACPR order, which was discussed with her family as a difficult and emotive decision.

Whilst receiving the ventilatory support, her respiratory pattern was seen to synchronise, and there was an improvement in her saturations. Furthermore, an assessment was made by intensivist specialists with paediatric respiratory, neonatal and neurology colleagues. A decision to wean her ventilatory oxygen support was made tentatively with careful monitoring of her respiratory pattern, observations and saturations. Satisfactory progress was being made, and neonatal specialists discussed the possibility of supplementary oxygen with her parents. The turnaround in her presentation allowed for discussion with the rehabilitation team, who suggested that after consistent positive observations on the intensive care unit that supplementary oxygen would be feasible in the community, and her DNACPR decision was revoked.

Her parents were taught how to practically use the home oxygen and given contacts for both the hospital and community rehabilitation teams with frequent follow-up. She remained under the joint care of paediatrics and respiratory specialists in the hospital with a direct phone contact in the case of desaturations and cyanosis or the patient becoming unwell, especially in the early days following discharge.

## 3. Discussion

The mechanism of central respiratory dysfunction in patients with Arnold–Chiari malformation is thought to be related to central herniation of intracranial contents with subsequent brainstem compression [8]. This can, in cases of respiratory centre compression, cause sudden loss of spontaneous respiratory output [8]. Alternatively, if the trigeminal nucleus or sensory nuclei are compressed, there can be cardiovascular compromise in the form of tachycardia and hypertension [8].

Our patient first underwent a lumbosacral myelomeningocele repair on Day 1 of life, then subsequently a VP shunt insertion, shunt revision and foramen magnum decompression with resection of a fourth ventricle choroid plexus papilloma, which may explain their respiratory dysfunction. Intervention from the surgery itself in the affected areas around the brainstem and craniocervical junction may have affected the respiratory function. Similarly, the change in pressure after the foramen magnum decompression itself could have led to changes in the nervous communication to the respiratory system. Interestingly, paediatric sleep studies revealed that REM sleep was associated with the apnoeic episodes. This finding suggests that other areas of the brain such as the tegmentum ‘the carpet' of the fourth ventricle, which is involved in dreaming and REM sleep, may have also been affected and negatively affected their breathing.

Other forms of respiratory dysfunction can be secondary to peripheral rather than central causes, such as upper airway obstruction secondary to vocal cord paralysis [9, 10]. Other presentations of neurological compromise in cases of Chiari malformation or myelomeningocele include orthostatic hypotension [11], bowel dysfunction [12], obstructive sleep apnoea [13] and as above vocal cord paralysis [9, 10].

Direct management of spina bifida, and specifically myelomeningocele, is a well-studied area of the literature, with the recent work such as the MOMS study looking into prenatal versus postnatal operative approaches and outcomes [14]. The study found benefit to myelomeningocele repair in utero and reduced incidence of hydrocephalus post birth compared to postbirth repair.

However, evidence to guide management on cases of respiratory dysfunction in patients with myelomeningocele and Arnold–Chiari malformation are much rarer. Isu et al. [15] report a case of bony foramen magnum decompression in a patient with Chiari malformation and respiratory distress with stridor, who recovered well from a respiratory perspective following a single surgical foramen magnum decompression, on a background of prior ventriculoperitoneal shunt insertion and myelomeningocele repair. This is in accordance with guidance from Sahu et al. [8], who observe that if a myelomeningocele has associated hydrocephalus present, a VP shunt should be inserted prior to myelomeningocele repair—whereas in the absence of hydrocephalus, the clinician may proceed directly to myelomeningocele repair [15].

In contrast, Atallah et al. review a case of respiratory arrest after posterior fossa decompression in a patient with Chiari I malformation [16]. Risk factors for a respiratory arrest after a posterior fossa decompression were identified as central respiratory centre dysfunction (which may be due to oedema and ischaemia), intraoperative brainstem ischaemia, abnormal respiratory mechanics and delayed gastric emptying [16]. Wan and Krassioukov [17] and Piper et al. [18] also emphasise the life-threatening complications of autonomic dysreflexia, including dependence on ventilators and death, highlighting the difficulties faced when managing such patients.

Another paper by Shellhaas et al. [19] comments on the association between Chiari II malformations and obstructive sleep apnoea or sleep-disordered breathing. Central hypoventilation disorders with apnoea can be acquired in cases of Arnold–Chiari malformation as well as in brain tumours or spinal injuries and can require oxygen therapy and ventilation if severe [20]. In Type II Arnold–Chiari malformation specifically, the causes of apnoea are both central as above or can be due to bilateral abductor cord paralysis and laryngomalacia [10, 21]. Much like the patient in our case, who experienced sleep-disordered breathing with recurrent episodes of central apnoea and a nocturnal BiPAP requirement, Shellhaas et al. observe that half of the Arnold–Chiari II patients experience sleep-disordered breathing due to a combination of the level of their spinal defect and brainstem and musculoskeletal abnormalities [19]. They call for an integrated approach to care for these complex cases across all relevant members of the multidisciplinary team. This is echoed by Shashidhar et al., who found that airway difficulties and autonomic dysfunction can complicate tonsillectomy cases for obstructive sleep apnoea in Arnold–Chiari malformation patients, and as such promote close working relations between neurologists, anaesthetists and the surgical team to minimise risk [13].

The above papers corroborate our own findings—in other words, myelomeningocele management is affected by the presence of hydrocephalus, and that central apnoea or hypoventilation syndromes can be a difficult presentation with life-threatening complications in patients with an Arnold–Chiari malformation. Our patient was palliated for 2 weeks following a respiratory arrest and repeated intubation, before recovering sufficiently to merit removal of their DNACPR form and return to full active management. The provision of care throughout the patient journey, from diagnosis, multiple operative interventions, respiratory support and repeated intubation, has required a cohesive effort between members of various medical and surgical specialities, and the multidisciplinary approach has allowed ongoing support to the point of becoming fit enough for a safe discharge home (Table 1). What this emphasises is that combining specialist input from different fields is tantamount, especially in the face of respiratory compromise as severe—and indeed near fatal—as this case.

## 4. Interdisciplinary Roles

Table 1shows the roles of the multidisciplinary team during the patient journey (own work).

## 5. Concluding Remarks

After medical investigation, it was found that urinary tract infections were triggering the patient's apnoeic episodes. Treating the urinary tract infections with antibiotics reduced these apnoeic episodes. The association between infection-causing stress on the body and autonomic dysfunction is evident in this case. The learning point is awareness that infections can act as trigger factors and affect respiratory function. The patient in the case was readmitted as an inpatient due to severe apnoea and hypoxia. Despite her young age of 6 months, she demonstrated resilience and was treated with noninvasive support overnight and oxygen when awake. It was during admission that the crucial linchpin association between urinary tract infections and apnoeic episodes were made and treated. One hypothesis is that the patient's autonomic respiratory dysreflexia was partly triggered by bladder irritation from infection on the background of damaged respiratory control centre sustained likely due to a combination of Chiari malformation, choroid plexus papilloma and surgical intervention around the region of the fourth ventricle.

Due to the observations of urinary tract infections triggering the patient's apnoeic episodes, we also take into consideration whether neonatal autonomic dysfunction may have been a unifying presentation which was not evident during her intensive care unit admission. Previously, the respiratory compromise diagnosed as an autonomic respiratory dysreflexia was not thought to involve the urinary system. However, the developments of urinary involvement suggest a more holistic picture of systemic dysregulation throughout the autonomic system of the neonate; therefore, ‘neonatal autonomic dysfunction' may well be a more appropriate term to describe this case. It may well be debated that the hypotension and desaturations observed as a sequalae of the reduced ability of brainstem coordination may have origins in the autonomic system.

## 6. Conclusion

Arnold–Chiari Type II malformation and myelomeningocele have a rare association with autonomic dysreflexia, including respiratory dysfunction. These patients can have a variety of outcomes, from transient episodes of apnoea through to dependence on ventilation and death.

This case highlights a rare case of profound respiratory dysfunction in which, with appropriate supportive care and involvement of the necessary MDT members, the patient was able to recover from a period of intubation in ITU and a prolonged period of nocturnal BiPAP requirement with supplemental daytime oxygen to discharge home.

The potential sequalae of foramen magnum decompression should prompt careful surgical planning and aftercare.

Careful consideration of management was given to this case, and her presentation improved from a near palliative state to one which was manageable with long-term respiratory support via tracheostomy. The decision to carry out a tracheostomy was made after extensive discussion and consideration of the patient's excellent neurodevelopmental progress .

Providing sufficient supportive care in the face of such cases is critical, and clinicians may consider a prolonged trial of supportive care before opting for a palliative approach, as progress can often be slow and gradual.

## Figures and Tables

**Table 1:** The roles of the multidisciplinary team during the patient journey.

Team	Role
Neurosurgery	Perform neurosurgical interventions including meningomyelocele repair, foramen magnum decompression, insertion of VP shunt and choroid plexus papilloma resection.
Paediatric respiratory medicine	Decisions surrounding weaning oxygen, monitoring respiratory function/sleep studies, intubation and tracheostomy.
Rehabilitation	Delivery of oxygen and community monitoring of respiratory saturations.
Neurology	Review of neurological function including assessing potential seizures
Neonatology	Managing apnoeic episodes, providing a ward environment as a main place of care for the patient.

## Data Availability

All data used in this report are from the patient's hospital notes, and therefore are not publicly available. Further enquiries can be directed to the corresponding author.
